# Prevalence and predictors of depressive and anxiety symptoms in a sample of women who use drugs in Tanzania: the key role of drug use stigma

**DOI:** 10.1186/s12888-023-05008-z

**Published:** 2023-07-18

**Authors:** Haneefa T. Saleem, Nora S. West, Samuel Likindikoki

**Affiliations:** 1 Bloomberg School of Public Health, Johns Hopkins University, Baltimore, MD USA; 2 School of Public Health and Social Sciences, Muhimbili University of Health and Allied Sciences, Dar es Salaam, Tanzania

**Keywords:** Depression, Anxiety, Women, Drug use stigma, Tanzania

## Abstract

**Background:**

Globally, women who use drugs face multiple health vulnerabilities, including poor mental health. Little is known about the mental health burden among women who use drugs in sub-Saharan Africa. This cross-sectional study examined the prevalence and predictors of depressive and anxiety symptoms among a sample of women who use drugs in Dar es Salaam, Tanzania.

**Methods:**

We administered a cross-sectional survey to a non-random sample of 200 women who use drugs in Dar es Salaam between November 2018 and March 2019. We used descriptive statistics to characterize the study sample and fitted separate logistic regression models to assess depressive and anxiety symptoms and their predictors.

**Results:**

The percentages of women reporting depressive and anxiety symptoms were 67.5% and 43.7%, respectively. Internalized drug use stigma (AOR = 1.34, 95% CI: 1.03–1.75) and prior attempts to stop heroin use (AOR = 2.99, 95% CI: 1.28-7.00) were associated with depressive symptoms. Enacted drug use stigma from health workers (AOR = 2.02, 95% CI: 1.34–3.04) and anticipated drug use stigma from family (AOR = 1.49, 95% CI: 1.02–2.16) were associated with anxiety symptoms.

**Conclusions:**

Depressive and anxiety symptoms were high among our study sample, with higher reports of symptoms of depression than anxiety. Drug use stigma was a key factor that contributed to elevated symptoms of depression and anxiety.

## Background

Poor mental health can adversely affect physical health and quality of life [1, 2]. Mental disorders are common among people who use drugs [3–5] and are known barriers to healthcare engagement [2]. Women who use drugs are particularly impacted by mental disorders. Globally, research has demonstrated that women who use drugs have high rates of mental disorders [6–8]. Women who use drugs often report high rates of post-traumatic stress disorder (PTSD), gender-based violence, and adverse childhood experiences, such as neglect and physical or sexual abuse, that are often internalized as anxiety, depression, and social withdrawal [9]. Much of what we know about mental disorders among people who use drugs, including women, is based on research conducted in high-income countries in North America, Europe, Oceania, and Asia [3, 9, 10]. Research on the mental health burden among people who use drugs in sub-Saharan Africa is limited, including in Tanzania where over 400,000 people use illicit drugs [11]. Mental health research on women who use drugs is even more limited, as women are underrepresented in substance use research in sub-Saharan Africa [12].

Several factors could affect rates of mental disorders among women who use drugs. Evidence suggests a bidirectional relationship between substance use severity and mental health with differences by gender [13, 14]. Previous research conducted in Tanzania has shown that women enrolled in opioid use disorder treatment had higher substance dependence and symptoms of depression and anxiety than their male counterparts [15]. Women who use drugs are more likely than men to use substances, including illicit drugs and alcohol, to self-medicate for underlying mental disorders [9]. In Tanzania, as in other settings, engagement in sex work can expose women who use drugs to physical and sexual violence [16, 17], which can exacerbate mental distress and contribute to mental disorders. For example, researchers have demonstrated an association between sexual violence and mental disorders, including drug dependence and suicidality, among women [18]. Furthermore, in Tanzania, where HIV prevalence is high among women who use drugs—estimates have ranged from 25 to 45% [19, 20]—, having a positive HIV diagnosis may negatively affect mental health, due to HIV-related stigma [21–23].

Drug use stigma can adversely impact the mental health of people who use drugs. Stigma related to illicit drug use has been associated with increased severity of problem substance use [24] and mental distress [25]. Drug use stigma comes from different sources, e.g., family, friends, colleagues and the source of stigma may have differential impacts on overall health and well-being [26, 27]. People who use drugs who experience discrimination are more likely to report mental distress, mental health problems, and have low mental functioning than those who do not report experiences of discrimination [28]. Women who use drugs may diverge in their experiences of stigma and discrimination compared to men who use drugs due to differences in gender roles and expectations, including perceptions of respectability [29]. Qualitative studies, mainly from North America, Oceania, and Asia, have indicated that women who use drugs experience greater drug use stigma than men [29]. Drug use stigma from healthcare providers can also negatively affect the mental health of people who use drugs. Healthcare providers and staff at health facilities may have negative attitudes towards patients who use drugs, perceiving violence, manipulation, and poor motivation as impeding their ability to deliver health care to these patients who use illicit drugs [30]. For example, in South Africa, people who use drugs have reported being made to feel guilt, shame, and loss of dignity due to discrimination from healthcare providers [31].

In Tanzania, women who use drugs face high disease burdens and vulnerabilities, including disproportionately high rates of HIV [19] and drug overdose risks [32]. Understanding the burden of mental disorders in women who use drugs and factors that might contribute to this burden is critical to mitigating the harmful effects of common mental disorders, such mood and anxiety disorders, to their physical health and quality of life. In this paper, we examine the prevalence and predictors of depressive and anxiety symptoms among a sample of women who use drugs, specifically heroin, in Dar es Salaam, Tanzania, with a particular focus on examining the relationship between drug use stigma and mental health outcomes.

## Methods

### Study participants and procedures

We administered a cross-sectional survey to 200 women who use drugs in Dar es Salaam between November 2018 and March 2019. Women were eligible to participate in the study if they were 18 years of age or older, reported heroin use in the past 30 days, resided in Dar es Salaam, and provided informed consent. Women were excluded from the study if they did not meet the inclusion criteria. We recruited participants via respondent-driven sampling techniques using initial seeds identified by community outreach workers who provide harm reduction services and resources for people who use drugs. Detailed study methods, including recruitment, have been previously reported [32]. Participants received three recruitment coupons to recruit eligible peers into the study. Participants were compensated 10,000 Tanzanian shillings (approximately 4.30 USD) to cover costs, i.e., travel and time, associated with completing the survey and received an incentive of 4,000 Tanzanian shillings (approximately 1.75 USD) for each eligible participant they recruited for the study. All participants provided oral informed consent prior to study participation. Oral consent was obtained because written consent would be the only record with the names of women participating in the study. Given the illegality of heroin use, women may have been hesitant to participate in a study if they knew that their names would be on record. Additionally, oral consent allowed us to mitigate risks of breaches in confidentiality that could have placed participants in trouble with law enforcement.

After obtaining informed consent, interviewers administered surveys in Swahili through face-to-face interviews using a structured questionnaire with responses recorded electronically on computer tablets. Quality control checks were built into the electronic survey. Survey interviewers were local women with university training in the social sciences and previous ethical research training who had extensive experience conducting surveys and qualitative interviews with vulnerable women and people who use drugs in Tanzania. Survey interviewers underwent a one-week study training focused on study-specific data collection methods and quality assurance. The structured survey questionnaire included questions about sociodemographic characteristics, social network characteristics, housing and activity spaces, drug and alcohol use, HIV prevention and treatment behaviors, drug use stigma, exposure to violence, and depression and anxiety. Interviewers informed participants that they could skip any question without penalty or stop the survey at any point. Surveys were conducted in a private room at a drop-in center for a community-based organization that provides harm reduction and social services for people who use drugs. Surveys took approximately 45 min to complete. The field data collection team debriefed daily, and the full study team met weekly to discuss recruitment, data collection, and data quality.

The study protocol was reviewed and approved by institutional review boards at the Muhimbili University of Health and Allied Sciences, the National Institute for Medical Research in Tanzania, and the Johns Hopkins University Bloomberg School of Public Health.

### Measures

#### Outcome variables

Our main outcome variables were depressive symptoms and anxiety symptoms. We assessed depressive symptoms using the Patient Health Questionnaire-9 (PHQ-9), in which scores range from 0 to 27, with higher scores indicating symptoms of more severe depression [33]. The scale had high internal consistency in our study sample (Cronbach’s alpha = 0.85). For our analyses, we dichotomized depressive symptoms scores using a cut-off of 9 for probable symptoms of depression; scores above 9 were used to indicate symptoms of a major depressive episode based on a previous validation study of the PHQ-9 conducted in Tanzania [34]. We assessed symptoms of anxiety using the Generalized Anxiety Disorder-7 (GAD-7) [35], in which scores range from 0 to 21, with higher scores indicating more severe symptoms of anxiety. The scale had high internal consistency in our study sample (Cronbach’s alpha = 0.89). We measured anxiety symptoms as a dichotomized variable (i.e., moderate to severe anxiety vs. minimum to mild anxiety) as scores greater than 9, consistent with recommended cut-offs [35] and as used in similar settings in East Africa [36, 37].

#### Predictor variables

Sociodemographic variables included age (continuous) and level of education (none, primary education, secondary education). HIV status was self-reported. We measured number of years of heroin use as a continuous variable. Daily heroin use was dichotomized into self-reported daily use versus less than daily use. Prior attempts to stop heroin use using any method, which could be considered an indication of substance use severity [38], was measured dichotomously (ever attempted to stop heroin use vs. never attempted to stop heroin use). We measured physical violence and sexual violence, specifically forced sex, in the past 12 months dichotomously.

We assessed drug use stigma using the SU-SMS, previously validated in samples of both in-treatment and out-of-treatment substance-using populations in the United States [39]. The SU-SMS was designed to capture substance use-related stigma across three stigma mechanisms (i.e., enacted, anticipated, and internalized) and two sources (i.e., family members and healthcare workers) using a total of 18 items. The original SU-SMS focused on substance use stigma more broadly, including alcohol and drugs in the language of survey items. We modified the language of the SU-SMS to limit the scale to assess stigma related to drug use only. The SU-SMS questionnaire was translated to Swahili and back-translated to English, reviewed by a Tanzanian psychiatrist with experience treating substance-using populations, and pre-tested with the target population to ensure interpretation and comprehension. Item responses were given on a 5-point Likert-type scale with higher scores indicating greater stigma. All subscales had high internal consistency: enacted drug use stigma from family (Cronbach’s alpha = 0.97), enacted drug use stigma from healthcare worker (Cronbach’s alpha = 0.95), anticipated drug use stigma from family (Cronbach’s alpha = 0.92), anticipated drug use stigma from healthcare workers (Cronbach’s alpha = 0.95), and internalized drug use stigma (Cronbach’s alpha = 0.90). We used mean scores of each subscale identified to create composite scores used in analyses; scores ranged from 0 to 4. The drug use stigma subscales were measured as continuous variables in models.

### Statistical analysis

We used descriptive statistics to characterize the study sample. We fitted separate unadjusted and adjusted logistic regression models for each mental health outcome. We used a p-value of < 0.05 and 95% confidence intervals to assess statistical significance in unadjusted and adjusted logistic regression models. Only variables with p-values of < 0.20 in unadjusted models were included in the multivariate-adjusted models.

Missing data were minimal. The proportion of missing data for drug use stigma items ranged from 0 to 4.5%. The proportion of missing data for depression and anxiety scales was 1.5% and 0.5%, respectively. Two covariates, physical violence in the past 12 months and forced sex in the past 12 months, had 1.5% and 3.5% missing data, respectively. We performed multiple imputations with chained equations to address missing data. We generated 10 imputed datasets to estimate parameters. To impute missing values, we specified the model with variables with missing values and auxiliary variables, including those that we intended to use in our model that did not have missing values (i.e., age, educational status, HIV status, relationship status, number of years of heroin use, daily heroin use, and having ever attempted to stop heroin use). We fitted logistic regression models on each imputed dataset, then pooled the results to get a single coefficient estimate for each variable in unadjusted and adjusted models.

All analyses, including imputations, were conducted using Stata/MP version 17.0 (StataCorp, College Station, Texas).

## Results

### Study sample characteristics

Characteristics of the full study sample are reported in Table 1. In summary, the mean age of participants was 33.5 years (standard deviation (SD) = 8.1 years, range: 19 to 56 years). Over a quarter (28%) of the study sample self-reported a positive HIV status. The mean number of years that women reported using heroin was 6.2 years (SD = 6.8 years, range: 0.5 to 33 years) and a majority (84.5%) reported daily heroin use. Almost two-thirds (61.9%) and more than a third (35.8%) of participants reported having experienced physical and sexual violence in the past 12 months, respectively. Drug use stigma subscale mean scores ranged from 1.0 for enacted drug use stigma from health workers to 3.0 for enacted drug use stigma from family. Anticipated drug use stigma from family and internalized drug use stigma mean scores were 2.4 and 2.2, respectively. Both enacted and anticipated drug stigma mean scores from health workers were lower at 1.0 and 1.3, respectively.

**Table 1:** Characteristics of women who use drugs included in the study (N = 200)

Characteristics	% (n)
Age in years, mean (range)	33.5 (19–56)
Educational level	
Primary school or less	86.0% (172)
Secondary school or higher	14.0% (28)
Self-reported HIV status	
HIV-negative	72.0% (144)
HIV-positive	28.0% (56)
Years spent using heroin, mean (range)	6.2 (0.5–33)
Frequency of heroin use	
Less than daily use	15.5% (31)
Daily use	84.5% (169)
Prior attempts to stop heroin use	
No	61.5% (123)
Yes	38.5% (77)
Experienced physical violence in past 12 months	
No	38.1% (75)
Yes	61.9% (122)
Experienced sexual violence in past 12 months	
No	64.2% (124)
Yes	35.8% (69)
Drug use stigma^a^, mean (SD)	
Enacted drug use stigma from family	3.0 (1.5)
Enacted drug use stigma from health workers	1.0 (1.5)
Anticipated drug use stigma from family	2.4 (1.2)
Anticipated drug use stigma from health workers	1.3 (1.3)
Internalized drug use stigma	2.2 (1.3)
Mental health	
Depressive symptoms	67.5% (133)
Anxiety symptoms	43.7% (87)

^a^ Drug use stigma scores range from 0 to 4 with higher values indicating greater stigma.

### Prevalence of depressive and anxiety symptoms

More than two-thirds (67.5%) and 43.7% of the study sample reported depressive and anxiety symptoms, respectively (Table 1).

### Predictors of depressive and anxiety symptoms

Table 2 presents results from unadjusted models for depressive and anxiety symptoms. All drug use stigma subscales were found to be significantly and positively associated with depressive symptoms in unadjusted models. Other variables found to be significantly associated with depressive symptoms included prior attempts to stop heroin use and experiencing physical and sexual violence in the past 12 months. Age, educational level, HIV status, years spent using heroin, and daily heroin use were not significantly associated with depressive symptoms in unadjusted models. All drug use stigma subscales, except internalized drug use stigma, were significantly associated with anxiety symptoms. Other predictors of anxiety symptoms found to be significant included years spent using heroin, daily heroin use, and experiencing sexual violence in the past 12 months.

**Table 2:** Unadjusted logistic regression models for depressive and anxiety symptoms among women who use drugs in Dar es Salaam, Tanzania (N = 200)

	Depressive Symptoms		Anxiety Symptoms	
Independent variable	Unadj. OR (95% CI)	p value	Unadj. OR (95% CI)	p value
Drug use stigma				
Enacted drug use stigma from family	1.26 (1.03–1.54)	**0.027**	1.64 (1.28–2.09)	**< 0.001**
Enacted drug use stigma from health workers	1.87 (1.38–2.55)	**< 0.001**	2.13 (1.66–2.74)	**< 0.001**
Anticipated drug use stigma from family	1.44 (1.11–1.86)	**0.007**	2.81 (1.96–4.03)	**< 0.001**
Anticipated drug use stigma from health workers	1.76 (1.35–2.30)	**< 0.001**	2.28 (1.77–2.95)	**< 0.001**
Internalized drug use stigma	1.54 (1.22–1.94)	**< 0.001**	1.20 (0.97–1.49)	0.091
Age	1.00 (0.97–1.04)	0.888	1.02 (0.99–1.06)	0.183
Educational level				
Primary school or less	ref		ref	
Secondary school or higher	1.25 (0.52-3.00)	0.625	1.52 (0.68–3.42)	0.311
Self-reported HIV status				
HIV-negative	ref		ref	
HIV-positive	1.18 (0.60–2.32)	0.627	1.02 (0.55–1.91)	0.946
Years spent using heroin	1.03 (0.99–1.09)	0.152	1.06 (1.01–1.10)	**0.011**
Frequency of heroin use				
Less than daily use	ref		ref	
Daily use	0.73 (0.31–1.76)	0.486	3.14 (1.28–7.68)	**0.012**
Prior attempts to stop heroin use				
No	ref		ref	
Yes	3.21 (1.62–6.35)	**0.001**	1.09 (0.61–1.94)	0.771
Experienced physical violence in past 12 months				
No	ref		ref	
Yes	1.98 (1.05–3.70)	**0.033**	1.79 (0.98–3.26)	0.057
Experienced sexual violence in past 12 months				
No	ref		ref	
Yes	3.39 (1.61–7.13)	**0.001**	2.30 (1.25–4.20)	**0.007**

Results of full adjusted models are presented in Table 3. In the full adjusted model for depressive symptoms, internalized drug use stigma was significantly associated with depressive symptoms after controlling for other covariates (AOR = 1.34, 95% CI: 1.03–1.75). Prior attempts to stop heroin use (AOR = 2.99, 95% CI: 1.28-7.00) was also statistically significant in the adjusted model for depressive symptoms. In the full adjusted model for anxiety symptoms, enacted drug use stigma from health workers (AOR = 2.02, 95% CI: 1.34–3.04) and anticipated drug use stigma from family (AOR = 1.49, 95% CI: 1.02–2.16) were the only variables found to be significantly associated with anxiety symptoms.

**Table 3:** Full adjusted logistic regression models for depressive and anxiety symptoms among women who use drugs in Dar es Salaam, Tanzania (N = 200)

Independent variable	Depressive Symptoms		Anxiety Symptoms	
	AOR (95% CI)	p value	AOR (95% CI)	p value
Drug use stigma				
Enacted drug use stigma from family	0.91 (0.70–1.18)	0.490	0.97 (0.71–1.32)	0.844
Enacted drug use stigma from health workers	1.52 (0.99–2.36)	0.057	2.02 (1.34–3.04)	**0.001**
Anticipated drug use stigma from family	1.24 (0.87–1.76)	0.239	1.49 (1.02–2.16)	**0.038**
Anticipated drug use stigma from health workers	1.24 (0.84–1.81)	0.275	1.34 (0.92–1.94)	0.124
Internalized drug use stigma	1.34 (1.03–1.75)	**0.030**	1.01 (0.76–1.33)	0.957
Years spent using heroin	0.96 (0.90–1.02)	--	1.01 (0.95–1.06)	0.861
Frequency of heroin use				
Less than daily use	ref		ref	
Daily use	--	--	2.35 (0.80–6.93)	0.122
Prior attempts to stop heroin use				
No	ref		ref	
Yes	2.99 (1.28-7.00)	**0.027**	--	--
Experienced physical violence in past 12 months				
No	ref		ref	
Yes	1.40 (0.66–2.92)	0.363	1.50 (0.68–3.30)	0.313
Experienced sexual violence in past 12 months				
No	ref		ref	
Yes	2.30 (0.98–5.36)	0.055	1.61 (0.71–3.69)	0.258

## Discussion

We examined the prevalence and predictors of depressive and anxiety symptoms among a sample of women who use drugs, specifically heroin, in Dar es Salaam, Tanzania. We demonstrated that the prevalence estimates of depressive and anxiety symptoms were high in the study sample; 67.5% and 43.7%, respectively. Internalized drug use stigma and prior attempts to stop heroin use were found to be independently and positively associated with depressive symptoms, while enacted drug use stigma from health workers and anticipated drug use stigma from family were independently and positively associated with symptoms of anxiety.

Drug use stigma was generally high in the study sample, possibly due to the illegality of heroin use and moral judgments that may be more pronounced among women who use drugs because of societal perceptions of deviation from gendered expectations of “respectability.” We found that the level of drug use stigma, however, varied by the source. Mean scores for enacted and anticipated drug use stigma from family as well as internalized drug use stigma were higher than enacted and anticipated stigma from health workers. It is possible that the lower scores for enacted and anticipated stigma from healthcare providers stem from less engagement with health services or behavior modification and non-disclosure of drug use in healthcare settings to actively avoid stigma [40]. The high report of drug use stigma from family is consistent with previous qualitative research conducted with women and men who use drugs in Tanzania, in which participants described stigma and mistrust from family and the detrimental impact on their well-being and health-seeking behaviors [41, 42]. Future research on other sources of drug use stigma that might be salient among women who use drugs and qualitative research that contextualizes their experiences of stigma from health workers is warranted.

Internalized drug use stigma was found to be significantly associated with depressive symptoms in the full adjusted model. There is strong evidence on the positive association between internalized substance use stigma, including drug use stigma, and depression [43–45], which our findings support. In addition to internalized stigma, the drug use stigma scale used in the present study focused on two main sources of enacted and anticipated stigma, from family and healthcare providers, which are stigma sources commonly identified in the literature [39]. We did not detect significant associations between these other stigma constructs and depressive symptoms in full adjusted models, despite research demonstrating a relationship between drug use stigma and depressive symptoms in other settings and for other substances. For example, a cross-sectional survey study conducted in Thailand with a non-random sample of 652 women with substance use disorder (with a majority reporting methamphetamine use) found an independent association between addiction stigma and depressive symptoms [46]. Another cross-sectional survey study from Taiwan conducted with a non-random sample of 255 men and 45 women with substance use disorders demonstrated a strong association between substance user stigma and depressive symptoms [47]. However, the divergence in results between our study and previous studies may be due to differences in setting, sample, and measures of stigma and depressive symptoms used. The relationship between enacted, anticipated, and internalized stigmas and depressive symptoms is complex. It is possible that we did not detect these associations because internalized drug use stigma partially mediated the relationship between enacted and anticipated drug use stigma and symptoms of depression. Previous research on mental illness stigma has shown associations between enacted stigma and internalized stigma [48, 49] and the mediation of this relationship by anticipated stigma [49, 50]. More research is needed on the relationship between stigma constructs and on the mediating role of internalized stigma in the relationship between enacted and anticipated stigmas and depression. Given the deleterious effects that depression can have on health-seeking and health-promoting behaviors, it is critical to implement interventions aimed at reducing drug use stigma, including internalized drug use stigma, and improving mental health.

Prior attempts to stop heroin use was also significantly associated with depressive symptoms in the full adjusted model. Though we did not screen for heroin dependence in the present study, unsuccessful attempts to stop using heroin could be interpreted as an indication of heroin dependence severity [38]. Researchers have shown that “recovered” individuals report lower levels of mental disorders [51]. The relationship between heroin dependence and depression is bidirectional: continued heroin use can result in depressive symptoms and depression may lead to continued heroin use [13, 14], this is especially true for women who are more likely than men to use substances to self-medicate [9] and more likely to suffer from internalizing problems such as depression and anxiety [15, 52]. Given the cross-sectional design of this study, we were unable to assess temporality or causality between heroin use and depressive symptoms, or heroin dependence severity and depressive symptoms. Regardless, in settings with limited resources for competing health priorities, targeting individuals who have made multiple prior attempts at stopping heroin use for mental health screening and services may be a feasible approach to identifying those with the greatest mental health care needs.

In the full adjusted model, enacted drug use stigma from health workers and anticipated drug use stigma from family were the only variables that we detected as significantly associated with anxiety symptoms. Enacted stigma from health workers and anticipated drug use stigma from family have been shown in previous research to contribute to anxiety [53, 54]. Enacted drug use stigma from health workers can adversely affect health care engagement [31] through anxiety, which can lead to the avoidance of health care [55] and poor health outcomes [56]. Anticipated drug use stigma from family may lead to avoidance of family and self-isolation, which could limit women’s access to social support critical for their physical and mental health and well-being [57, 58]. Interventions that address stigma against people who use drugs at multiple levels, including the family and health care system levels, might reduce anxiety among this population and, ultimately, support treatment-seeking and care engagement [59, 60]. For example, at the health system level, training for health providers on substance use disorders might help to reduce their stigmatizing attitudes about people who use drugs [60]. Additionally, peer-delivered services or peer navigation support in health care settings could help to mitigate the effects of stigma on women who use drugs [61]. At the family level, communication strategies that promote better understanding of substance use disorders and motivational interviewing with the families of people who use drugs might improve families’ attitudes toward loved ones struggling with a substance use disorder [60].

Despite their significant associations in unadjusted models, we did not detect associations between variables for years spent using heroin and frequency of heroin use nor sexual violence, and symptoms of anxiety in full adjusted models. The longer time since drug use initiation and the frequency of drug use might be associated with more severe drug dependence and higher drug use stigma from multiple sources, so drug use stigma may have confounded their relationship with anxiety symptoms in the adjusted model. Unexpectedly, reported experience of sexual violence was also not observed to be statistically significantly associated with symptoms of anxiety in the final adjusted model, which warrants further investigation.

### Limitations

This study has limitations. First, the cross-sectional study design did not allow for assessment of temporality or causality in the relationship between independent variables and the mental health outcomes in our study. Second, though our use of respondent-driven sampling techniques to recruit women who use drugs was effective with this hidden population, it is inherently non-probabilistic and thus our results are not generalizable to the population of women who use drugs in Dar es Salaam. We may have oversampled women with more severe problematic heroin use and those involved in sex work, who may be more likely to have a history of trauma [16] and, thus, higher reports of depressive and anxiety symptoms compared to women with less severe heroin use, which could have resulted in selection bias. Even so, women with more severe heroin use are in most need of being reached with interventions that can connect them to mental health and substance use disorder treatment. Third, surveys were completed in 45 min, on average. Though we used face-to-face survey administration, which has been shown to present low cognitive burden on survey respondents [62], it is possible that the quality of responses to survey items may have decreased over the administration of the survey presenting potential response biases [63]. Fourth, there were likely unmeasured confounding variables that we did not account for in our analyses. For example, we did not measure engagement with the healthcare system in the survey questionnaire, beyond HIV care enrollment for those participants self-reporting living with HIV, nor did our measure of drug use stigma from healthcare providers distinguish between different types of health services (e.g., general, HIV care, harm reduction), potentially a source of measurement bias. We also did not include a measure of drug use disclosure in healthcare settings or among families. It is possible that the lower reports of enacted and anticipated stigma from healthcare providers might be due to limited interactions with the health system and stigma scores from family might be influenced by whether family members were aware of the respondents’ drug. Therefore, the relationship between drug use stigma from healthcare providers and family and our outcomes of depression and anxiety may have been attenuated in our analyses. Finally, our sample size, specifically our relatively small sample of women who use drugs with HIV (56 out of 200), precluded us from adopting an intersectional approach to our analyses by examining interactions between drug use stigma and HIV stigma and their joint effects on mental health for the subsample with HIV [64]. Relatedly, we did not include measures for sex work stigma or HIV stigma, which have both been shown to be associated with poor mental health [65]. Examining the intersection between drug use stigma, sex work stigma, and HIV stigma, as well as the influence of healthcare engagement across different types of health services (e.g., HIV, substance use disorder treatment, etc.) on experiences of stigma, will be important areas for future research on women who use drugs in this and similar settings.

## Conclusion

Women who use drugs in Tanzania report high depressive and anxiety symptoms, which can negatively affect their physical health and well-being and their ability to seek and access needed health services, including harm reduction services. Drug use stigma is a key factor that contributes to elevated symptoms of depression and anxiety witnessed in this population. Interventions aimed at addressing the high burden of depressive and anxiety symptoms among women who use drugs and reducing drug use-related stigma will be important for reducing the harmful effects of mental disorders on the overall health and well-being of this vulnerable population.

## Data Availability

The aggregate data analyzed during the current study are available from the corresponding author on reasonable request.
